# Discovery of Novel Viruses in *Culicoides* Biting Midges in Chihuahua, Mexico

**DOI:** 10.3390/v16071160

**Published:** 2024-07-19

**Authors:** S. Viridiana Laredo-Tiscareño, Javier A. Garza-Hernandez, Chandra S. Tangudu, Wichan Dankaona, Carlos A. Rodríguez-Alarcón, Jaime R. Adame-Gallegos, Erick J. De Luna Santillana, Herón Huerta, Rodolfo Gonzalez-Peña, Alejandra Rivera-Martínez, Ezequiel Rubio-Tabares, Diana M. Beristain-Ruiz, Bradley J. Blitvich

**Affiliations:** 1 Department of Veterinary Microbiology and Preventive Medicine, College of Veterinary Medicine, Iowa State University, Ames, IA 50011, USA; viridiana.laredo@gmail.com (S.V.L.-T.); ctangudu@iastate.edu (C.S.T.); retro_89@hotmail.com (W.D.); 2 Laboratorio Entomología Médica, Instituto de Ciencias Biomédicas, Universidad Autónoma de Ciudad Juárez, Ciudad Juárez, Chihuahua 31125, Mexico; biolgarza@gmail.com (J.A.G.-H.);; 3 Animal Virome and Diagnostic Development Research Unit, Department of Pathology, Faculty of Veterinary Science, Chulalongkorn University, Bangkok 10330, Thailand; 4 Departamento de Ciencias Veterinarias, Instituto de Ciencias Biomédicas, Universidad Autónoma de Ciudad Juárez, Ciudad Juárez, Chihuahua 32310, Mexico; carrodri@uacj.mx (C.A.R.-A.); diana.beristain@uacj.mx (D.M.B.-R.); 5 Facultad de Ciencias Químicas, Universidad Autónoma de Chihuahua, Chihuahua 32310, Mexico; jadame@uach.mx; 6 Laboratorio Medicina de la Conservación, Centro de Biotecnología Genómica del Instituto Politécnico Nacional, Reynosa, Tamaulipas 88700, México; edeluna@ipn.mx; 7 Laboratorio de Entomología, Instituto de Diagnóstico y Referencia Epidemiológicos, Ciudad de México 01480, Mexico; cerato_2000@yahoo.com; 8 Laboratorio de Arbovirología, Centro de Investigaciones Regionales “Dr. Hideyo Noguchi”, Universidad Autónoma de Yucatán, Mérida, Yucatan 97225, Mexico

**Keywords:** *Culicoides*, midges, virus discovery, metagenomics, RNA-seq, Mexico

## Abstract

Biting midges (*Culicoides*) are vectors of many pathogens of medical and veterinary importance, but their viromes are poorly characterized compared to certain other hematophagous arthropods, e.g., mosquitoes and ticks. The goal of this study was to use metagenomics to identify viruses in *Culicoides* from Mexico. A total of 457 adult midges were collected in Chihuahua, northern Mexico, in 2020 and 2021, and all were identified as female *Culicoides reevesi*. The midges were sorted into five pools and homogenized. An aliquot of each homogenate was subjected to polyethylene glycol precipitation to enrich for virions, then total RNA was extracted and analyzed by unbiased high-throughput sequencing. We identified six novel viruses that are characteristic of viruses from five families (*Nodaviridae*, *Partitiviridae*, *Solemoviridae*, *Tombusviridae,* and *Totiviridae*) and one novel virus that is too divergent from all classified viruses to be assigned to an established family. The newly discovered viruses are phylogenetically distinct from their closest known relatives, and their minimal infection rates in female *C. reevesi* range from 0.22 to 1.09. No previously known viruses were detected, presumably because viral metagenomics had never before been used to study *Culicoides* from the Western Hemisphere. To conclude, we discovered multiple novel viruses in *C. reevesi* from Mexico, expanding our knowledge of arthropod viral diversity and evolution.

## 1. Introduction

Biting midges (genus *Culicoides*, family *Ceratopogonidae*) are the most abundant hematophagous insects worldwide, with a geographic distribution encompassing the tropics, subtropics, tundra, and temperate regions [1,2,3]. Many female *Culicoides* require blood meals for egg production, and their bites are often painful and sometimes cause acute allergic reactions. Moreover, *Culicoides* are vectors of many viruses, bacteria, parasitic protozoa, and nematodes of medical and veterinary importance. An example of a *Culicoides*-transmitted viral pathogen in humans is the Oropouche virus (family *Peribunyaviridae*) [4,5]. The Oropouche virus occurs in Central and South America, where it has caused over half a million cases of febrile illness, with some cases accompanied by aseptic meningitis. *Culicoides*-transmitted viruses of veterinary importance include the Akabane virus (family *Peribunyaviridae*), the African horse sickness virus (AHSV; family *Sedoreoviridae*), the bluetongue virus (BTV; family *Sedoreoviridae*), the bovine ephemeral fever virus (BEFV; family *Rhabdoviridae*), and the Schmallenberg virus (family *Peribunyaviridae*) [1,6,7,8,9,10]. *Culicoides*-transmitted viruses that cause wildlife disease include AHSV, BTV, BEFV, and epizootic hemorrhagic disease virus (family *Sedoreoviridae*) [11].

The advent of rapid and inexpensive unbiased high-throughput sequencing platforms and bioinformatics tools has resulted in the discovery of numerous viruses that would have been difficult, if not impossible, to detect using traditional virus detection techniques [12,13,14,15]. These technologies have allowed for the detection of novel viruses in diverse sample types (animal, plant, and environmental). Numerous viral metagenomics studies have been performed on hematophagous arthropods, but most have focused on mosquitoes and ticks, with *Culicoides* spp. midges and other hematophagous arthropods being relatively neglected [16,17,18,19,20,21].

A small number of studies have characterized the viromes of *Culicoides* spp. midges [22,23,24,25,26,27,28]. Viromes have been characterized for *C. arakawae* from Japan, *C. imicola* from Senegal, *C. impunctatus* from Scotland, at least three *Culicoides* spp. (*C. arakawae*, *C. lungchiensis*, and *C. punctatus*) from Zhoushan Island in China, an unspecified number of *Culicoides* spp. from Yunnan in China, and ten *Culicoides* spp. from Greece. Many taxonomically diverse viruses were identified in these studies. For example, 14 novel viruses from at least 10 families were detected in the midges from Greece [22]. However, the viromes of *Culicoides* spp. midges from the Western Hemisphere have not been characterized. In this study, a metagenomics-based approach was used to determine the composition and diversity of viruses in *C. reevesi* from Mexico.

## 2. Materials and Methods

### 2.1. Study Sites and Midge Collections

**Figure 1:**
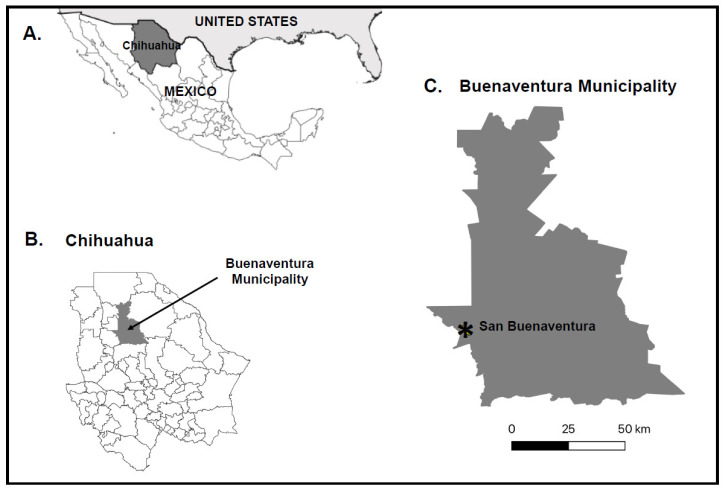
Geographic locations of the collection sites. (**A**) State map of Mexico, with Chihuahua shaded dark gray, (**B**) municipality map of Chihuahua, with Buenaventura municipality shaded dark gray, and (**C**) Buenaventura municipality, with the city of San Buenaventura denoted by an asterisk.

Study sites were established close to San Buenaventura, a town in the municipality of Buenaventura in Chihuahua, northern Mexico (Figure 1). Collections were performed in 2020 and 2021 along the Santa Maria River, which borders San Buenaventura to the west. Blood-seeking midges were collected from 4.00 to 8.00 p.m. using the human landing catch method. Hand-held aspirators were used to remove midges from the exposed arms of the collectors before blood meals could be acquired. Midges were placed into individual cryostorage vials and transported in liquid nitrogen to the laboratory at the Universidad Autónoma de Ciudad Juárez. Midges were placed on chill tables, then inspected under a microscope and morphologically identified using published taxonomic keys [29,30,31]. Midges were sorted into pools of up to 100 individuals and stored at −80 °C until they were transported on dry ice by express delivery to Iowa State University.

### 2.2. Homogenizations

Midges were placed in polypropylene, round-bottom 5 mL tubes with 2 mL of phosphate-buffered saline (PBS) supplemented with 100 units/mL penicillin and 100 μg/mL streptomycin. Four 4.5-mm-diameter copper-clad steel beads (BB-caliber airgun shot) were added to each tube and midge pools were homogenized by vortexing for 30 s. Midge homogenates were centrifuged (10,000× *g*, 10 min, 4 °C) and supernatants were collected and stored at −80 °C.

### 2.3. Polyethylene Glycol Precipitation

An aliquot of each supernatant was subjected to polyethylene glycol (PEG) precipitation to enrich for virions. Briefly, 100 μL of each supernatant was added to 900 μL of PBS then centrifuged (10,000× *g*, 10 min, 4 °C) to remove any residual debris. Supernatants were filtered, mixed with an equal volume of 2 × PEG solution (20% PEG-8000 [*w*/*v*] and 0.6M NaCl in PBS, pH 7.4) and rotated overnight at 4 °C. Samples were centrifuged (15,000× *g*, 10 min, 4 °C) and pellets were resuspended in PBS.

### 2.4. Unbiased High-Throughput Sequencing

Unbiased high-throughput sequencing (UHTS) was performed as previously described, with minor modifications [32]. Briefly, total RNA was extracted from each PEG-precipitated sample using Trizol Reagent (ThermoFisher Scientific, Wattham, MA, USA). An aliquot was taken from each total RNA sample then the aliquots were mixed together to create a single sample. Ribosomal RNA was depleted from the sample using the NEBNext^®^ rRNA Depletion Kit (New England BioLabs, Ipswich, MA, USA), and an RNAseq library was generated using the NEBNext^®^ Ultra™ II Directional RNA Library Prep Kit for Illumina^®^ (New England BioLabs). Sequencing was performed using the Novaseq 6000 system (Illumina, San Diego, CA, USA) at the Iowa State University DNA Facility.

### 2.5. Bioinformatics

Sequencing reads were analyzed using the FastX Toolkit (http://hannonlab.cshl.edu/fastx_toolkit/) (accessed on 1 December 2023) to remove barcodes and low-quality ends (Phred quality score ≥ 33). Duplicate reads were identified and removed using Cdhit-454 (http://weizhongli-lab.org/cd-hit/) (accessed on 1 December 2023). Host sequences were depleted by mapping the remaining reads to the genome of *Culicoides sonorensis* using Bowtie 2 [33]. *Culicoides sonorensis* is the only *Culicoides* spp. with a fully sequenced genome [34]. Unmapped reads were analyzed using the sortMeRNA program to remove ribosomal RNA-related reads [35]. Remaining reads were subjected to de novo SPAdes assembly (version 3.5.0) [36]. Contigs were aligned by BLASTn, BLASTx, and tBLASTx to the NCBI nucleotide database (downloaded December 2023) using an e-value of <10^−5^. Unaligned contigs were translated into all six reading frames and matches were found using BLASTp and InterProScan 5 [37]. Data were transformed by Python programming (https://www.python.org/).

### 2.6. RT-PCR and Sanger Sequencing

RT-PCRs were performed to retrospectively identify the pools that contained the newly discovered viruses and to confirm the species identity of midges. Complementary DNAs were generated using Superscript III reverse transcriptase (ThermoFisher Scientific) and PCRs were performed using high-fidelity *Taq* polymerase (Thermo Fisher Scientific) in accordance to the manufacturer’s instructions. Primers specific to the newly discovered viruses were designed from the sequences generated by UHTS. For midges species confirmation, two primer pairs were used: universal primers that amplify a 710 bp region of the invertebrate mitochondrial cytochrome c oxidase subunit I (COI) gene [38] and primers designed in-house using a *C. reevesi* voucher COI gene sequence of 496 bp from the Genbank database (forward primer: 5′-GATTAGTTCCCCTTATACTCGG-3′; reverse primer: 5′-AAAATATAAACTTCTGGATGTCC-3′). RT-PCR products were purified using the PureLink gel extraction kit (ThermoFisher Scientific) and sequenced using a 3730x1 DNA Analyzer (Applied Biosystems, Foster City, CA, USA) at the Iowa State University DNA Facility.

### 2.7. Virus Isolation in Cell Culture

We attempted to isolate each novel virus by performing virus isolation using *Aedes albopictus* (C6/36) mosquito and African green monkey kidney (Vero) cells. *Culicoides* cell lines have been developed [39] but none are commercially available. C6/36 cells were cultured in Liebovitz L15 medium (Thermo Fisher Scientific) and Vero cells were cultured in Dulbecco’s modified Eagle medium (Thermo Fisher Scientific). All media was supplemented with a 10% fetal bovine serum (FBS), 2 mM of L-glutamine, 100 units/mL of penicillin, and 100 μg/mL of streptomycin, except when cultures needed to be maintained with minimal cell proliferation, in which case, the concentration of FBS was reduced to 2%. C6/36 cells were cultured at 28 °C and Vero cells were cultured at 37 °C with 5% CO_2_. Homogenates were filtered and inoculated onto subconfluent monolayers of C6/36 or Vero cells in 75 cm^2^ flasks. The cells were incubated for 1 h at room temperature on an orbital shaker, then the media was removed. Cells were rinsed five times in PBS and incubated in 12 mL of fresh media for 7 days. Supernatants were collected and an aliquot (100 μL) of each supernatant was inoculated onto new subconfluent monolayers of the same cell type. The process was repeated until three cell culture passages had been performed. Total RNA was extracted from the final passage supernatants and tested for viral RNA by RT-PCR.

### 2.8. Phylogenetic Analysis

Amino acid sequences were aligned using MUSCLE [40]. Bayesian phylogenetic trees were constructed using BEASTv1.10.4 [41]. Phylogenies were performed under the WAG amino acid substitution model with Gamma + Invariant sites using 4 as the number of gamma categories, an uncorrelated relaxed clock model with lognormal relaxed distribution and a constant-size coalescent priors while sampling across the sites for 10 million sampling iterations, discarding the first 25% as burn-in. Midpoint-rooted tree figures were created using Figtree. Select nodes are labeled with posterior probability values.

## 3. Results

### 3.1. Midge Collections and Virus Identification

A total of 457 adult midges were collected in Chihuahua, morphologically identified as female *Culicoides reevesi*, and sorted into five pools. Species identifications were confirmed by amplifying and sequencing a region of the COI gene using primers designed in-house because the universal primers did not generate amplicons. RT-PCR products were sequenced, and the resulting sequences were aligned and revealed to have 100% nucleotide identity with each other. Because the COI sequences are identical, only one was deposited into the Genbank database (Genbank Accession No. PP359630). Our sequences have 98.4% nucleotide identity to the corresponding regions of *C. reevesi* voucher COI gene sequences previously deposited into the Genbank database.

Unbiased high-throughput sequencing revealed that the midges contained seven novel viruses but no previously known viruses (Table 1). Five viruses could be assigned to four established families (*Nodaviridae*, *Partitiviridae*, *Tombusviridae*, and *Totiviridae*), and another belongs to the family *Solemoviridae*, or a closely related, but yet-to-be-created family. The final virus was too divergent from all classified viruses to be assigned to an established family. The library contained 11,449,252 high-quality reads, deposited into the NCBI database under Biosample Accession No. PRJNA1127052. After non-viral reads were subtracted, 12,980 viral reads remained. The average read depth of each novel virus is provided (Table S1). Each pool was retrospectively analyzed by RT-PCR using virus-species primers to identify those that contained novel viruses (Figure S1). The minimal infection rates (MIRs) in female *C. reevesi* for the novel viruses ranged from 0.22 to 1.09 (Table 2). None of the viruses replicated in C6/36 or Vero cells.

### 3.2. Nodaviridae

The family *Nodaviridae* has two recognized genera (*Alphanodavirus* and *Betanodavirus*) which consist of viruses that infect insects and fish, respectively [42]. Many noda-like viruses and unclassified nodaviruses (e.g., nodaviruses not formally assigned to a genus) have been detected in other metazoans, most notably crustaceans and nematodes [43,44,45,46,47]. Nodaviruses have bipartite, positive-sense RNA genomes of 3.1 kb (RNA1) and 1.4 kb (RNA2) that encode an RNA-dependent RNA polymerase (RdRp) and capsid protein precursor, respectively [42].

We provide evidence of a novel nodavirus, designated Chihuahua culicoides nodavirus 1 (CCNV1). A 996 nt region of the CCNV1 genome was recovered (Genbank Accession No. PP101790) and it encodes a predicted 289-residue translation product characteristic of a capsid protein truncated at the C-terminus (Table 1 and Table S2). The translation product has greatest (34.5%) amino acid identity (98% coverage) to the corresponding region of an unclassified virus listed in the Genbank database as *Riboviria* sp., which was detected in an anal swab collected from a Radde’s warbler (*Phylloscopus schwarzi*) in China (no article available, Genbank Accession No. QJI53480.1). Alignments were also performed using the predicted translation product of CCNV1 and the corresponding regions of a representative virus from each established genus of the family *Nodaviridae*. The CCNV1 translation product has 21.0% identity (100% coverage) to the black beetle virus (an alphanodavirus) and 23.6% identity (100% coverage) to the barfin flounder nervous necrosis virus (a betanodavirus), while the two classified viruses have 23.5% identity (100% coverage) to one another.

**Figure 2:**
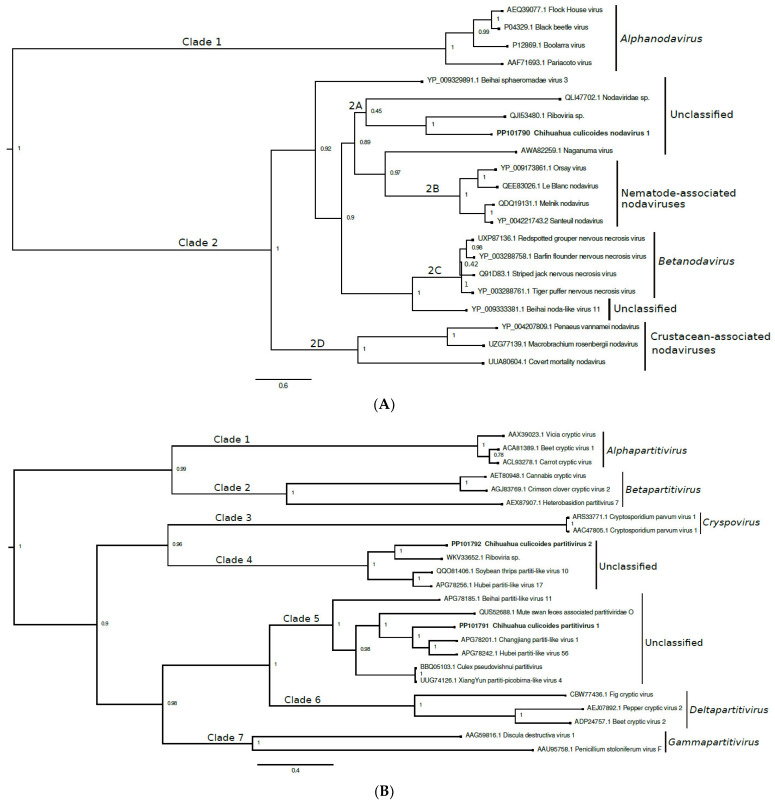

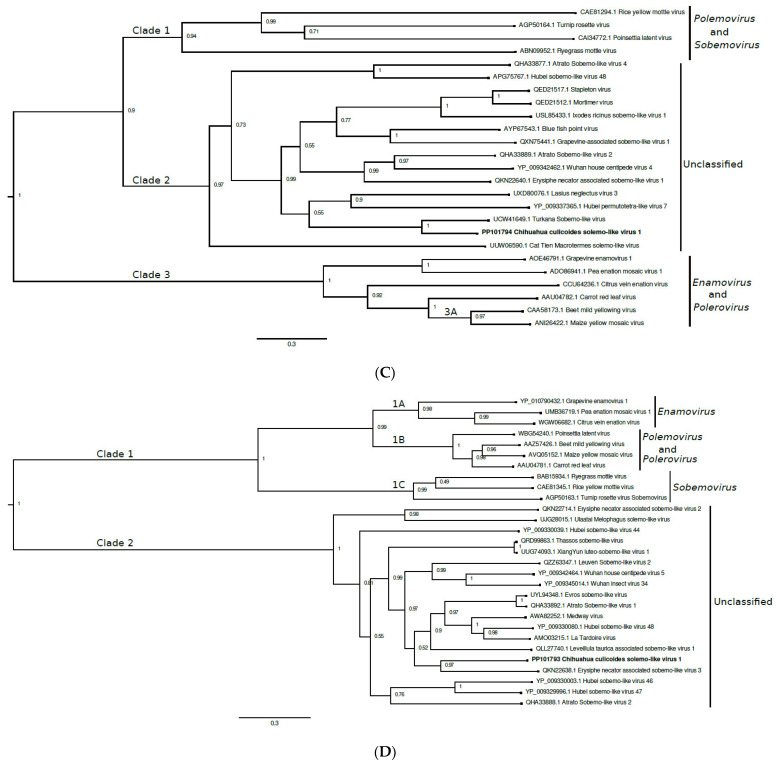

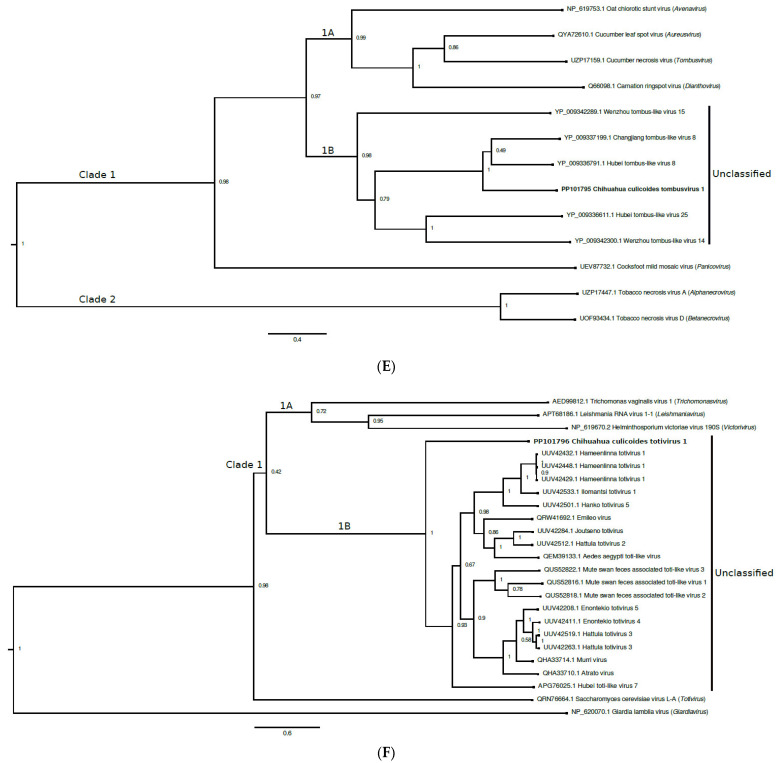
Phylogenetic relationships among each novel virus found in this study and closely related viruses from the taxonomic groups (**A**) *Nodaviridae*, (**B**) *Partitiviridae*, (**C**,**D**) *Solemoviridae*, (**E**) *Tombusviridae*, and (**F**) *Totiviridae*. Amino acid sequences were aligned using MUSCLE and Bayesian phylogenetic trees were constructed using BEASTv1.10.4. Select nodes are labeled with posterior probability values. Viruses identified in this study are bolded. Select genus names are denoted in parentheses.

Bayesian inference was used to analyze the partial capsid protein sequences of CCNV1 and select closely related viruses (Figure 2A). Two distinct clades (denoted as clades 1 and 2) are observed and the posterior support for each grouping is 1.0. Clade 1 comprises classified viruses of the genus *Alphanodavirus*. Clade 2 contains four nested clades (denoted as 2A to 2D). CCNV1 is in clade 2A, along with two unclassified viruses detected in avian swabs. The posterior support for this topological arrangement is not strong (0.45). Clades 2B, 2C, and 2D contain nematode-associated nodaviruses, betanodaviruses, and crustacean-associated nodaviruses, respectively. We propose that CCNV1 should be classified within the family *Nodaviridae* and assigned to a yet-to-be-established genus.

### 3.3. Partitiviridae

The family *Partitiviridae* has five recognized genera (*Alphapartitivirus*, *Betapartitivirus*, *Gammapartitivirus*, *Deltapartitivirus*, and *Cryspovirus*) and consists of viruses that infect plants, fungi, and protozoa [48]. Unclassified partitiviruses and partiti-like viruses have been detected in other metazoans, including *Culicoides* spp. midges [22,47,49,50,51]. Partitiviruses have bipartite, double-stranded RNA genomes of 3.0 to 4.8 kbp (1.4 to 2.4 kbp per segment) [48]. The genomic segments are designated dsRNA1 and dsRNA2 and encode the RdRp and capsid protein, respectively.

We detected two novel viruses, designated Chihuahua culicoides partitivirus 1 and 2 (CCPV1 and CCPV2, respectively). A 1543 bp region of the CCPV1 genome was sequenced (Genbank Accession No. PP101791), and it contains a complete open reading frame (ORF) predicted to encode a 497-residue RdRp. The translation product has the greatest (67.8%) amino acid identity (99% coverage) to the corresponding region of Hubei partiti-like virus 56, an unclassified partiti-like virus detected in insects (unspecified species) in China [47]. We also sequenced a 1703 bp region of the CCPV2 genome (Genbank Accession No. PP101792). The sequence encodes a predicted 561-residue protein characteristic of an RdRp truncated at the N-terminus. The translation product has the greatest (62.3%) amino acid identity (95% coverage) to the corresponding region of an unclassified virus, designated Riboviria sp., detected in an anal swab collected from a bird (unspecified species) in China (no article available, Genbank Accession No. WKV33652.1). The CCPV1 and CCPV2 amino acid sequences have 30.0% identity (54% coverage) to each other and ≥21.5% amino acid identity (≥30% coverage) to the corresponding regions of select classified viruses in the family *Partitiviridae* (Table S3).

A phylogenetic tree was constructed using the partial RdRp sequences of CCPV1, CCPV2, and select closely related viruses (Figure 2B). Viruses from all five established genera of the family *Partitiviridae* were included. Seven clades are observed (denoted as clades 1 to 7). The posterior support for each grouping is ≥0.96. CCPV1 and CCPV2 are in clades 5 and 4, respectively. Both clades comprise unclassified viruses detected in insects, crustaceans, and/or avian swabs and feces. Clades 1, 2, 3, 6, and 7 contain viruses belonging to the genera *Alphapartitivirus*, *Betapartitivirus*, *Cryspovirus*, *Deltapartitivirus*, and *Gammapartitivirus*, respectively. We propose that the family *Partitiviridae* requires two new genera, one to accommodate CCPV1 and the other clade 5 viruses, and the second for CCPV2 and its clade 4 counterparts.

### 3.4. Solemoviridae

The family *Solemoviridae* consists of viruses with single-stranded, positive-sense RNA genomes of 4 to 6 kb, and some of these viruses are important pathogens of crops [52]. The family contains four established genera: *Enamovirus*, *Polemovirus*, *Polerovirus*, and *Sobemovirus*. These viruses are usually transmitted via mechanical wounding, vegetative propagation, or insects (i.e., aphids and beetles). Unclassified solemoviruses and solemo-like viruses have also been described, some of which were detected in *Culicoides* spp. midges [22,23]. Solemo-like viruses with bipartite genomes of 4.1 to 4.6 kb (1.5 to 2.8 kb per segment) have also been described, with many detected in insects [22,47,53].

We provide evidence of a novel virus species, designated as Chihuahua culicoides solemo-like virus 1 (CCSV1), which is closely related to viruses in the family *Solemoviridae*. Two contigs of 1159 and 1480 were detected (Genbank Accession Nos. PP101793-4, respectively). The 1480 nt. contig contains a complete ORF that encodes a predicted 216-residue capsid protein (Table 1). The translation product has greatest (59.0%) identity (100% coverage) to the putative capsid protein of Turkana Sobemo-like virus, an unclassified virus detected in midges (unspecified species) in Kenya (no article available, Genbank Accession No. UCW41649.1). Turkana Sobemo-like virus has a bipartite genome. The 1159 nt. contig contains a partial ORF predicted to encode a 383-residue RdRp truncated at the N-terminus. The translation product has greatest (78.8%) identity (55% coverage) to the partially sequenced RdRp of Turkana Sobemo-like virus, but a higher BLAST E-value and greater coverage (57.4% identity and 97% coverage) with the putative RdRp of Erysiphe necator-associated sobemo-like virus 3, a sobemo-like virus detected in fungus in Spain (no article available, Genbank Accession No. QKN22638.1).

A phylogenetic tree was constructed using the capsid protein sequence of CCSV1 and the corresponding regions of select closely related viruses (Figure 2C). Erysiphe necator-associated sobemo-like virus 3 was not included because its capsid protein gene has not been sequenced. Three clades are observed (denoted as clades 1 to 3). Clade 2 contains CCSV1 and other unclassified solemo-like viruses. The posterior support for this grouping is 0.97. Clade 1 contains viruses from the genus *Sobemovirus*, in addition to Poinsettia latent virus, the sole member of the genus *Polemovirus* [52]. Poinsettia latent virus is a recombinant virus, with the 5′ three-quarters of its genome closely related to the corresponding region of poleroviruses and its capsid protein sequence closely related to those of sobemoviruses [54]. Clade 3 contains enamoviruses and poleroviruses, with the latter forming a nested clade (denoted as 3A).

Another tree was constructed using the partial RdRp sequence of CCSV1 and the corresponding regions of select closely related viruses (Figure 2D). Turkana Sobemo-like virus was not included because its RdRp gene is unresolved at the 3′ end and the sequence in the Genbank database is considerably shorter than the sequences used for the analysis. Two major clades are observed (denoted as clades 1 and 2), and the posterior support for both groupings is 1.0. CCSV1 is in clade 2, which comprises unclassified solemo-like viruses. CCSV1 is closely related phylogenetically to Erysiphe necator associated sobemo-like virus 3. Clade 1 contains three nested clades (denoted as 2A to 2C). Clades 2A and 2C contain viruses in the genera *Enamovirus* and *Sobemovirus*, respectively, while clade 2B contains viruses in the genus *Polerovirus* in addition to the Poinsettia latent virus, the sole member of the genus *Polemovirus*.

We propose that at least one new genus needs to be created to accommodate CCSV1 and the other unclassified solemo-like viruses, but it is ambiguous whether these viruses belong to the family *Solemoviridae* or to a closely related, yet-to-be-established family. In the phylogenetic tree constructed using RdRp sequences, CCSV1 and the other unclassified solemo-like viruses belong to a different clade than the classified solemoviruses, with neither clade basal to the other, making the family designation of CCSV1 unclear. However, in the phylogenetic tree created using capsid protein sequences, the viruses in clade 3 (enamoviruses and poleroviruses) are basal to those in clade 1 (unclassified solemo-like viruses) and clade 2 (polemoviruses and sobemoviruses), suggesting that it is not necessary to create a new family.

### 3.5. Tombusviridae

The family *Tombusviridae* contains 18 genera of plant viruses, most of which have monopartite positive-sense RNA genomes of 3.7 to 4.8 kb, although some have bipartite genomes [55]. Tombusviruses are usually spread by mechanical transmission, seed and pollen transmission, and through infected plant material used for propagation and grafting, and sometimes by fungal and beetle vectors. Many unclassified tombusviruses and tombus-like viruses have been detected in other metazoans, including midges [17,23,49,56].

We identified a novel virus, designated Chihuahua culicoides tombusvirus 1 (CCTV1), an apparent member of the family *Tombusviridae*. A 327 nt region of the CCTV1 genome was sequenced (Genbank Accession No. PP101795) and it encodes a predicted 108-residue translation product characteristic of a capsid protein truncated at both termini. The translation product has greatest (50.5%) amino acid identity (87% coverage) to the corresponding region of an unclassified tombus-like virus, designated as Hubei tombus-like virus 8, discovered in a mixed pool of insects from China [47]. When compared to classified viruses, CCTV1 has greatest (37.8%) amino acid identity (32% coverage) to Oat chlorotic stunt virus (genus *Avenavirus*), a soil-borne virus of cereals [57].

Bayesian inference was used to analyze the partial capsid protein sequences of CCTV1 and select other viruses, including viruses from seven of the 18 genera of the family *Tombusviridae* (Figure 2E). The viruses grouped into two main clades (denoted as clades 1 and 2), with clade 1 containing two nested clades (denoted as 1A and 1B). CCTV1 has a close phylogenetic relationship with Changjiang tombus-like virus 8 and Hubei tombus-like virus 8, unclassified tombus-like viruses detected in crustaceans and insects, respectively [47]. These viruses are in clade 1B, along with other unclassified tombus-like viruses. The posterior support for this topological arrangement is 0.98. Clades 1A and 2 contain classified tombusviruses. We propose that a new genus needs to be created within the family *Tombusviridae* to accommodate CCTV1.

### 3.6. Totiviridae

The family *Totiviridae* contains five recognized genera (*Giardiavirus, Leishmaniavirus, Totivirus, Trichomonasvirus,* and *Victorivirus*) [55,58,59]. Viruses in the genera *Totivirus* and *Victorivirus* mostly infect fungi and yeast, while those in the genera *Giardiavirus*, *Leishmaniavirus*, and *Trichomonasvirus* infect parasitic protozoa. Many unclassified totiviruses and toti-like viruses have recently been detected in arthropods, bats, crustaceans, fish, and plants [50,60,61,62,63,64]. Viruses in the family *Totiviridae* have monopartite, double-stranded RNA genomes of 4.6 to 7.0 kbp that contain two overlapping ORFs. The 5′-proximal ORF encodes the capsid protein and several additional proteins while the 3′-proximal ORF encodes the RdRp.

We sequenced a 774 bp region of the genome of a novel virus, designated Chihuahua culicoides totivirus 1 (CCTotiv1; Genbank Accession No. PP101796). The deduced amino acid sequence encodes a predicted 258-residue translation product characteristic of an RdRp truncated at both termini. The predicted translation product has greatest (40.0%) amino acid identity (100% coverage) to the corresponding region of mute swan feces associated toti-like virus 1, an apparent totivirus identified in avian fecal material in the United Kingdom (no article available, Genbank Accession No. QUS52816.1). Pairwise alignments revealed that the CCTotiv1 sequence has 22.9 to 35.2% identity (≥40% coverage) to the corresponding regions of the type virus species of each genus of the family *Totiviridae* (Table S4). The type species have 20.2 to 31.2% identity (≥8% coverage) to one other.

Bayesian inference was used to analyze the partial RdRp sequences of CCTotiv1 and select closely related viruses, including a representative virus from each of the five genera of the family *Totiviridae* (Figure 2F). Most viruses are grouped within a large clade (denoted as clade 1), but the posterior support for this topological arrangement (0.4) is not strong. Clade 1 contains two nested clades (1A and 1B). Clade 1B consists exclusively of unclassified toti-like viruses and includes CCTotiv1. The posterior support for this topological arrangement is 1.0. Clade 1A consists of three viruses: Helminthosporium victoriae virus 190S, Leishmania RNA virus 1-1, and Trichomonas vaginalis virus 1, which belong to the genera *Victorivirus*, *Leishmaniavirus*, and *Trichomonasvirus*, respectively. Basal to clade 1 are Saccharomyces cerevisiae virus L-A and Giardia lamblia virus (genera *Totivirus* and *Giardiavirus*, respectively). We propose that at least one new genus needs to be created within the family *Totiviridae* to accommodate CCTotiv1 and the other clade 1B viruses.

### 3.7. Unclassified Virus

We detected a novel virus, designated Chihuahua culicoides virus 1 (CCV1), which could not be assigned to an established family because it is too divergent from all classified viruses. A 1849 nt region of the CCV1 genome was sequenced (Genbank Accession No. PP101797) and shown to contain one complete and one partial ORF. The complete ORF is predicted to encode a 532-residue protein of unknown function that has the greatest (25.2%) identity (94% coverage) to the corresponding region of Leuven wasp-associated virus 1, an unclassified virus detected in wasps from Belgium [65]. The partial ORF is predicted to encode a 389-residue protein characteristic of an RdRp truncated at the C-terminus. The translation product has the greatest (36.8%) identity (100% coverage) to the corresponding region of Leuven wasp-associated virus 1. Both CCV1 translation products have no significant identity to any unclassified viruses. Phylogenetic trees were not generated because the phylogenies would not be of assistance in the family-level classification of CCV1.

## 4. Discussion

We report the detection of multiple novel RNA viruses in *C. reevesi* from Mexico. The viruses are taxonomically diverse, belonging to multiple established families (*Nodaviridae*, *Partitiviridae*, *Tombusviridae*, *Totiviridae*, and possibly *Solemoviridae*) or being too divergent from all classified viruses to be assigned to an established family. Two novel viruses were detected in all five pools, indicating that they commonly infect *C. reevesi* in the study area, although a larger number of midges needs to be tested to accurately estimate the viral MIRs. No previously known viruses were detected, but this was not unexpected because viral metagenomics had never before been performed on *C reevesi* or any other *Culicoides* spp. midges from the Western Hemisphere. All previous studies were performed on midges from countries in the Eastern Hemisphere, namely China, Greece, Japan, Kenya, Scotland, and Senegal [22,23,24,25,26,27,28].

Isolates were not obtained for any viruses. These experiments were performed using C6/36 and Vero cells. A *Culicoides* cell line was not used because none are commercially available, even though they have been developed for *C. nubeculosus*, *C. sonorensis*, and *C. variipennis* [39,66,67]. A likely explanation why no viruses were isolated is because they have narrow host-ranges that preclude mosquito and vertebrate cell replication. A less likely explanation is that none of the midges contained an infectious virus, despite the maintenance of a continuous cold-chain. In this regard, viral nucleic acid is more stable than infectious virions. In all other *Culicoides* metagenomics studies, virus isolation was not attempted using any eukaryotic cell lines [22,23,24,25,26,27,28], although giant viruses were isolated from *C. imicola* in Senegal using amoebal cultures [27].

The closest known relative of each novel virus was determined by BLAST analysis and Bayesian inference. In each case, the closest known relative was a poorly characterized virus detected in insects or avian swabs. It is unknown whether the viruses detected in swabs replicate in birds or were acquired through the consumption of virus-infected material (i.e., insects). None of the closest relatives are known to replicate in humans, vertebrate animals or plants and our phylogenetic data indicate that most, if not all, of the newly discovered viruses are insect-specific. Some insect-specific viruses (ISVs) are capable of modulating the replication and transmission of pathogenic viruses. Therefore, ISVs may affect human and vertebrate animal health despite their insect-only phenotypes [68,69,70,71,72,73]. Dengue virus 1 (a pathogenic flavivirus) is transmitted to mice more efficiently by mosquitoes also infected with Phasi Charoen-like virus (an insect-specific phasivirus) and Humaita Tubiacanga virus (an unclassified ISV) compared to mosquitoes not infected with these ISVs [69]. Eilat virus (an insect-specific alphavirus) delays the dissemination of chikungunya virus (a pathogenic alphavirus) in mosquitoes [68]. Experiments have not been performed to determine whether *Culicoides*-associated ISVs modulate the replication or transmission of pathogenic viruses.

Based on the genomic organizations of their closest known relatives, four viruses detected in our study likely have bipartite genomes. The viruses are CCNV1 (a nodavirus), CCPV1 and CCPV2 (both partitiviruses), and CCSV1 (a solemo-like virus). A characteristic feature of nodaviruses and partitiviruses is the presence a bipartite genome, where RNA1 encodes the RdRp and RNA2 encodes the capsid protein [42,48]. Solemoviruses have monopartite genomes, but a rapidly increasing number of solemo-like viruses with bipartite genomes have been described [22,47,52,53]. We detected both genomic segments of CCSV1, but only RNA1 of the partitiviruses and RNA2 of CCNV1. One explanation why only RNA1 was detected for the partitiviruses is because the RdRp is the most conserved protein of RNA viruses, making RdRp sequences the easiest viral sequences to identify during the bioinformatics analysis [74,75,76]. There are many other examples of unclassified nodaviruses where sequence data are available only for the RdRp-encoding segment [77,78,79,80,81]. As noted above, only RNA2 was detected for CCNV1. RdRp sequences were undoubtedly present, but potentially at levels below our detection limit. Sequences encoding the capsid protein, but not the RdRp, were also recovered for the Barns Ness breadcrumb sponge noda-like virus 1, an unclassified nodavirus discovered in a sea sponge off the coast of Scotland [82].

## 5. Conclusions

We report the discovery of multiple novel viruses in *Culicoides* biting midges from Mexico. These findings provide new insights into the diversity, host range, phylogeny, and taxonomy of arthropod-associated viruses. These findings also add to the rapidly growing plethora of viruses discovered in recent years using unbiased high-throughput sequencing and bioinformatics.

## Figures and Tables

**Table 1:** Novel viruses detected in midges from Mexico and their proposed taxonomic classification and closest known relatives.

Virus	Proposed Taxonomic Classification	^1^ Amount of Genome Sequenced (nt. or bp)	^2^ Closest Known Relative Based on Amino Acid Sequence Alignments	% Amino Acid Identity (% Coverage) [Translation Product(s)]
Chihuahua culicoides nodavirus 1	*Nodaviridae*	996	*Riboviria* sp. (QJI53480.1)	34.5 (98) [CP]
Chihuahua culicoides partitivirus 1	*Partitiviridae*	1543	Hubei partiti-like virus 56 (APG78242.1)	67.8 (99) [RdRp]
Chihuahua culicoides partitivirus 2	*Partitiviridae*	1703	*Riboviria* sp. (WKV33652.1)	62.3 (95) [RdRp]
Chihuahua culicoides solemo-like virus 1	*Solemoviridae*?	1159 [segment 1]	Erysiphe necator associated sobemo-like virus 3 (QKN22638.1)	57.4 (99) [RdRp]
		1480 [segment 2]	Turkana Sobemo-like virus (UCW41649.1)	59.0 (100) [CP]
Chihuahua culicoides tombusvirus 1	*Tombusviridae*	327	Hubei tombus-like virus 8 (YP_009336791.1)	50.5 (87) [CP]
Chihuahua culicoides totivirus 1	*Totiviridae*	774	Mute swan feces associated toti-like virus 1 (QUS52816.1)	40.0 (100) [RdRp]
Chihuahua culicoides virus 1	Unclassified	1849	Leuven wasp-associated virus 1 (QZZ63336.1, QZZ63337.1)	25.2 (94) [HP] 36.8 (100) [RdRp]

CP, capsid protein; HP, hypothetical protein; RdRp, RNA-dependent RNA polymerase. ^1^ For each virus, only the longest contig was deposited into the GenBank database, unless the virus has a segmented genome, in which case the longest contig for each segment was deposited. ^2^ GenBank Accession No. of closest known relatives are provided in parentheses.

**Table 2:** Minimal infection rates in female *Culicoides reevesi* for the novel viruses.

Virus	^a^ No. Pools Positive	^b^ Minimal Infection Rate
Chihuahua culicoides nodavirus 1	1	0.22
Chihuahua culicoides partitivirus 1	5	1.09
Chihuahua culicoides partitivirus 2	3	0.66
Chihuahua culicoides solemo-like virus 1	5	1.09
^c^ Chihuahua culicoides tombusvirus 1	1	0.22
Chihuahua culicoides totivirus 1	3	0.66
Chihuahua culicoides virus 1	1	0.22

^a^ There are a total of five pools; ^b^ Calculated as (the number of positive pools divided by the total number of midges tested) × 100; ^c^ CCTV1 RNA was detected by RT-PCR in the single PEG-precipitated sample, but none of the five pools comprising this sample, even though three primer pairs were used. One explanation for this finding is the amount of viral RNA is below the limit of detection of the RT-PCR, unless PEG precipitation is used to remove the non-viral RNA and concentrate the viral RNA. We have assumed that at least one pool contains CCTV1 RNA.

## Data Availability

The data presented in this study are available in the present article and the GenBank database (accession numbers PP101790-7 and PP359630); biosample accession No. PRJNA1127052.

## Supplementary Materials

The following supporting information can be downloaded at: https://www.mdpi.com/article/10.3390/v16071160/s1, Figure S1. Retrospective analysis of midge pools by RT-PCR using primers specific to (A) Chihuahua culicoides nodavirus 1, (B), Chihuahua culicoides partitivirus 1, (C), Chihuahua culicoides partitivirus 2, (D) Chihuahua culicoides solemo-like virus 1, (E) Chihuahua culicoides totivirus 1, and (F) Chihuahua culicoides virus 1. Reactions were performed using total RNA extracted from the five pools (lanes 1-5). A non-template negative control was also included (lane 6). M denotes the molecular weight marker with the size of each band denoted. RT-PCRs were also performed using Chihuahua culicoides tombusvirus 1-specific primers, but no amplicons were detected and therefore, the gel image is not shown; Table S1: Average read depths of novel viruses detected in midges from Mexico; Table S2: Predicted genome sizes and percentage of genome coverage of novel viruses detected in midges from Mexico; Table S3: Genetic relatedness between Chihuahua culicoides partitiviruses 1 and 2 and select classified viruses within the family *Partitiviridae*; Table S4: Genetic relatedness between Chihuahua culicoides totivirus 1 and the type species of each genus within the family *Totiviridae*.
