# Supplementary material for: Discovery of Novel Viruses in Culicoides Biting Midges in Chihuahua, Mexico
Source: Viruses. 2024 Jul 19;16(7):1160. doi: 10.3390/v16071160 (PMC11281482; doi:10.3390/v16071160)
Supplement: Supplementary file 1 [file viruses-16-01160-s001.zip › viruses-3063655-supplementary.pdf]

Table S1. Average read depths of novel viruses detected in midges from Mexico

Virus	Average read depth
Chihuahua culicoides nodavirus 1	20.8
Chihuahua culicoides partitivirus 1	827.7
Chihuahua culicoides partitivirus 2	158.7
Chihuahua culicoides solemo-like virus 1	128.7
Chihuahua culicoides tombusvirus 1	8.9
Chihuahua culicoides totivirus 1	19.9
Chihuahua culicoides virus 1	26.2

Table S2. Predicted genome sizes and percentage of genome coverage of novel viruses detected in midges from Mexico

Virus	Amount of genome sequenced (nt. or bp)	[a] Estimated genomic length (kb or kbp)	Estimated genomic coverage (%)
Chihuahua culicoides nodavirus 1	996	4.5	22
Chihuahua culicoides partitivirus 1	1543	3.0 - 4.8	32 - 51
Chihuahua culicoides partitivirus 2	1703	3.0 - 4.8	35 - 57
Chihuahua culicoides solemo-like virus 1	2639	4.1 - 4.6	57 - 64
Chihuahua culicoides tombusvirus 1	327	3.7 - 4.8	7 - 9
Chihuahua culicoides totivirus 1	774	4.6 - 7.0	11 - 17
Chihuahua culicoides virus 1	1849	3.7	50

[a] Based on genomic sequence data available for closely related viruses

Table S3. Genetic relatedness between Chihuahua culicoides partitiviruses 1 and 2 and select classified viruses within the family *Partitiviridae*

Virus (genus)	[1] % Amino acid identity (% coverage)	
	Virus	
	Chihuahua culicoides partitivirus 1	Chihuahua culicoides partitivirus 2
Beet cryptic virus 1 (*Alphapartitivirus*)	26.7 (37)	24.7 (54)
Carrot cryptic virus (*Alphapartitivirus*)	26.8 (37)	23.6 (52)
Cannabis cryptic virus (*Betapartitivirus*)	24.3 (52)	24.4 (65)
Heterobasidion partitivirus 7 (*Betapartitivirus*)	21.5 (58)	26.5 (53)
Cryptosporidium parvum virus 1 (*Cryspovirus*)	31.1 (30)	27.6 (63)
Beet cryptic virus 2 (*Deltapartitivirus*)	31.4 (76)	25.0 (55)
Fig cryptic virus (*Deltapartitivirus*)	29.8 (87)	25.1 (73)
Penicillium stoloniferum virus F (Gammapartitivi-rus)	27.6 (66)	26.3 (74)
Discula destructiva virus 1 (*Gammapartitivirus*)	30.6 (89)	31.3 (52)

[1] Sequence alignments were performed using the 497- and 561-residue translation products of Chihuahua culicoides partitiviruses 1 and 2, respectively and the corresponding regions of various other partitiviruses. Genbank Accession nos. are provided in Figure 2B.

Table S4. Genetic relatedness between Chihuahua culicoides totivirus 1 and the type species of each genus within the family *Totiviridae*

Virus (Genus)	[1]% Amino acid identity (% coverage)					
	Virus					
	CCTotiv1	GLV	HvV190S	LRV-1-1	ScV-L-A	TVV-1
Chihuahua culicoides totivirus 1	N/A					
Giardia lamblia virus (*Giardiavirus*)	22.9 (50)	N/A				
Helminthosporium victoriae virus 190S (*Victorivirus*)	30.2 (52)	21.7 (12)	N/A			
Leishmania RNA virus 1-1 (*Leishmaniavirus*)	35.2 (40)	25.4 (9)	30.2 (83)	N/A		
Saccharomyces cerevisiae virus L-A (*Totivirus*)	27.3 (51)	20.2 (16)	25.9 (47)	27.7 (47)	N/A	
Trichomonas vaginalis virus 1 (*Trichomonasvirus*)	34.3 (40)	27.0 (8)	31.2 (54)	27.1 (53)	26.4 (33)	N/A

[1] Sequence alignments were performed using the 258-residue translation product of Chihuahua culicoides totivirus 1 and the corresponding regions of the various other totiviruses; CCTotiv1: Chihuahua culicoides totivirus 1; GLV: Giardia lamblia virus; HvV190S: Helminthosporium victoriae virus 190; N/A: not applicable; ScV-L-A: Saccharomyces cerevisiae virus L-A; TVV-1: Trichomonas vaginalis virus 1. Genbank Accession nos. are provided in Figure 2F.

Figure S1. Retrospective analysis of midge pools by RT-PCR using primers specific to (A) Chihuahua culicoides nodavirus 1, (B), Chihuahua culicoides partitivirus 1, (C), Chihuahua culicoides partitivirus 2, (D) Chihuahua culicoides solemo-like virus 1, (E) Chihuahua culicoides totivirus 1, and (F) Chihuahua culicoides virus 1. Reactions were performed using total RNA extracted from the five pools (lanes 1-5). A non-template negative control was also included (lane 6). M denotes the molecular weight marker with the size of each band denoted. RT-PCRs were also performed using Chihuahua culicoides tombusvirus 1-specific primers, but no amplicons were detected and therefore, the gel image is not shown.

A.

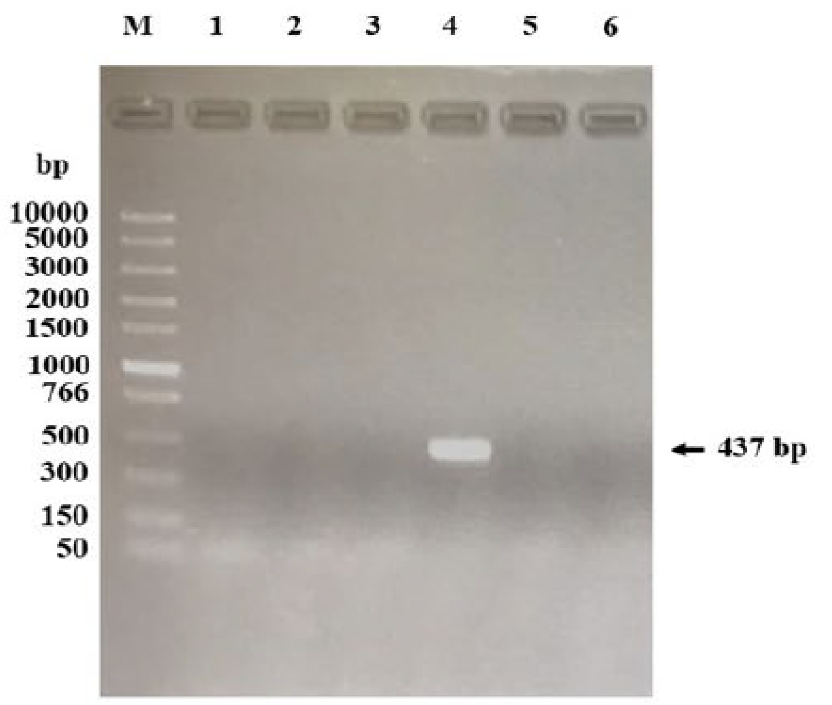

B.

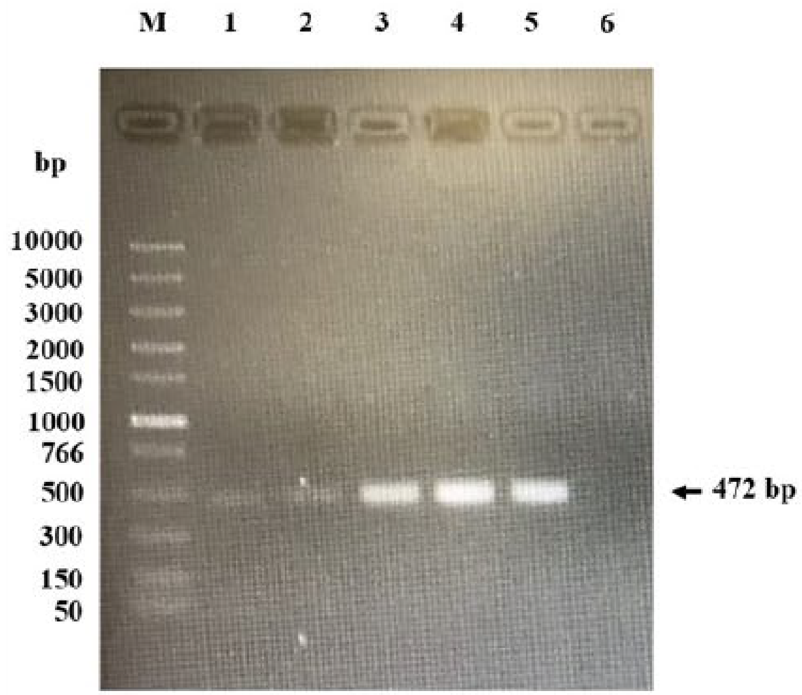

C.

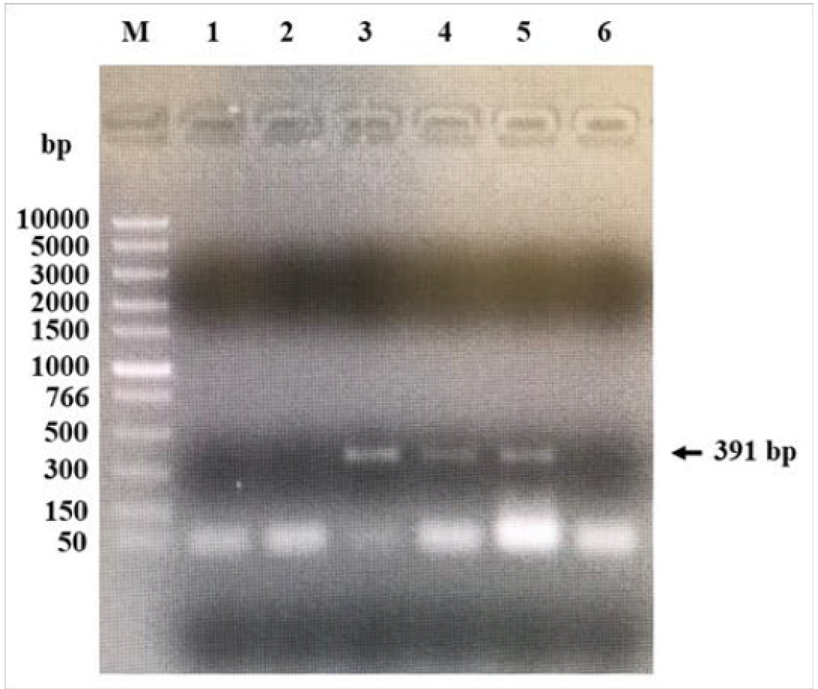
M 1 2 3 4 5 6
bp
10000
5000
3000
2000
1500
1000
766
500
300
150
50
← 391 bp

D.

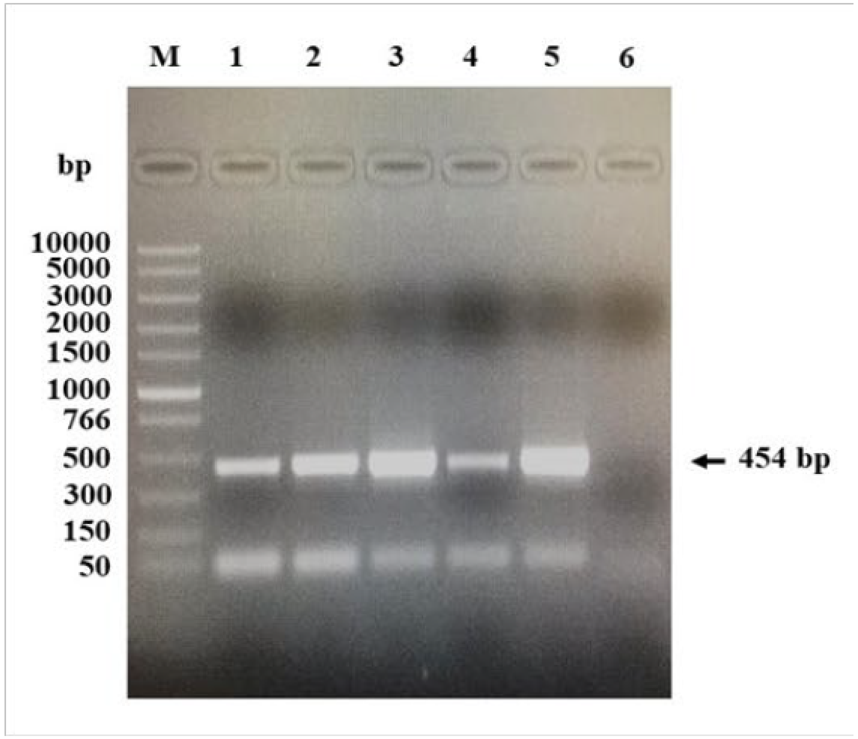
M 1 2 3 4 5 6
bp
10000
5000
3000
2000
1500
1000
766
500
300
150
50
← 454 bp

E.

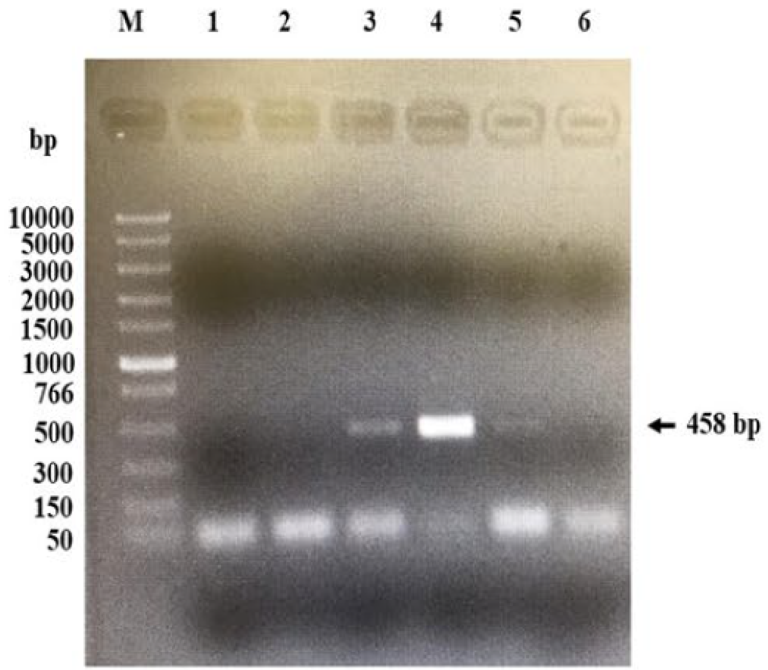
M 1 2 3 4 5 6
bp
10000
5000
3000
2000
1500
1000
766
500
300
150
50
458 bp

F.

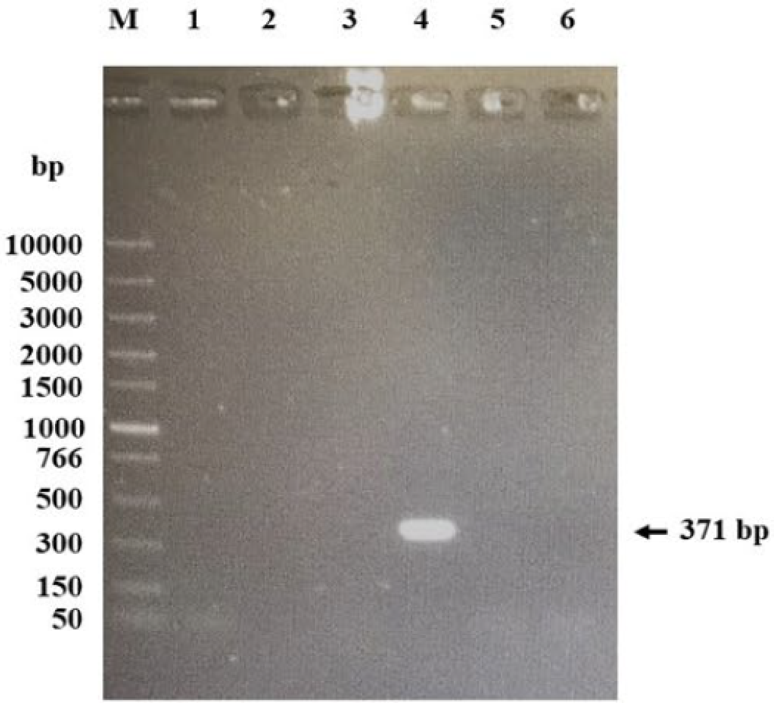
M 1 2 3 4 5 6
bp
10000
5000
3000
2000
1500
1000
766
500
300
150
50
371 bp
